# Temperature-Dependent Luminescence of Red-Emitting Ba_2_Y_5_B_5_O_17_: Eu^3+^ Phosphors with Efficiencies Close to Unity for Near-UV LEDs

**DOI:** 10.3390/ma13030763

**Published:** 2020-02-07

**Authors:** Egle Ezerskyte, Julija Grigorjevaite, Agne Minderyte, Sebastien Saitzek, Arturas Katelnikovas

**Affiliations:** 1 Institute of Chemistry, Faculty of Chemistry and Geoscience, Vilnius University, Naugarduko 24, LT-03225 Vilnius, Lithuania; egle.ezerskyte@chf.stud.vu.lt (E.E.); julija.grigorjevaite@chf.vu.lt (J.G.); agne.minderyte@chf.stud.vu.lt (A.M.); 2 Université d’ARTOIS, CNRS, Centrale Lille, ENSCL, Université de Lille, UMR 8181, Unité de Catalyse et Chimie du Solide (UCCS), F-62300 Lens, France

**Keywords:** near-UV LED, borate, Eu^3+^, red phosphor, ceramics, CIE 1931 colour coordinates, luminous efficacy, thermal quenching, quantum efficiency

## Abstract

Solid state white light sources based on a near-UV LED chip are gaining more and more attention. This is due to the increasing efficiency of near-UV-emitting LED chips and wider phosphors selection if compared to devices based on blue LED chips. Here, a brief overview is given of the concepts of generating white light employing near-UV LED and some optical properties of the available phosphors are discussed. Finally, the synthesis and optical properties of very efficient red-emitting Ba_2_Y_5_B_5_O_17_:Eu^3+^ phosphor powder and ceramics is reported and discussed in terms of possible application as a red component in near-UV LED-based white light sources.

## 1. Introduction

**Figure 1:**
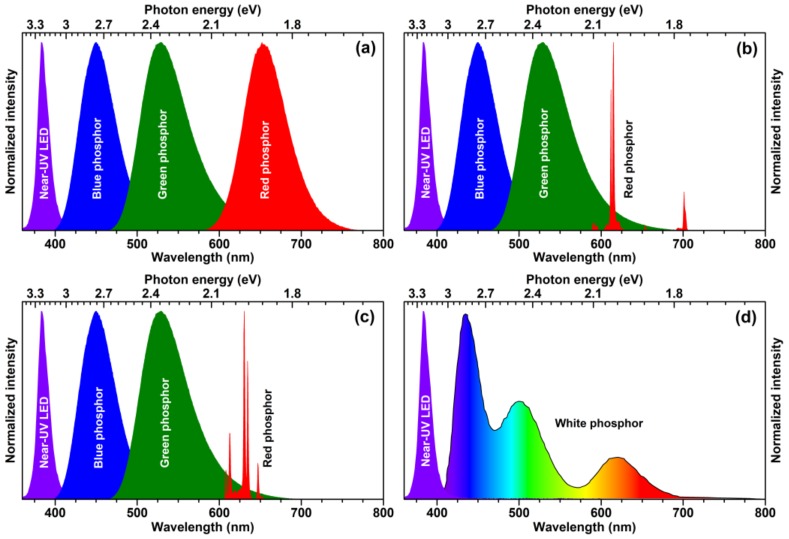
Normalized emission spectra of near-UV LED (λ_em_ = 380 nm) based white light sources with all broadband-emitting phosphors (**a**), line-emitting red phosphor (**b**,**c**) and single-phase white-light-emitting phosphor (**d**). Here blue phosphor is BaMgAl_10_O_17_:Eu^2+^; green—Ba_2_SiO_4_:Eu^2+^; red—Li_3_Ba_2_Eu_3_(MoO_4_)_8_ (**b**) and K_2_SiF_6_:Mn^4+^ (**c**); white—Ba_3_MgSi_2_O_8_:Eu^2+^, Mn^2+^ (**d**).

During the past decade, phosphor-converted white light-emitting diodes (pc-WLEDs) were the research interest for many scientists and engineers because of their outstanding brightness and luminous efficiency, low power consumption, long device lifetime, reliability and eco-friendly characteristics compared to other lighting sources, for instance, incandescent and halogen light bulbs, and fluorescent lamps. To date, white LEDs are the most efficient lighting source [1,2,3]. There are several ways to produce white light by employing a blue or near-UV-emitting LED chip. The first white LEDs were prepared by putting the yellow-emitting phosphor on the blue-emitting LED chip; however, such approach yielded cool white light with low colour rendering index (CRI) thus unsuitable for indoor lighting. In order to solve the deficiency of intensity in the red spectral region, the red-emitting phosphor was added together with yellow-emitting phosphor and the CRI and correlated colour temperatures (CCT) of obtained white light sources were significantly improved. At the very beginning of the solid state lighting (SSL) research the efficiency of near-UV-emitting LED chips was considerably lower if compared to their blue-emitting counterparts; therefore, they were not considered for application in SSL sources. However, with constant development of LED chips, the efficiency of near-UV-emitting LED chips was significantly improved and this opened a new possibility to generate white light. The graphical representation of several ways to produce white light employing the near-UV LED is given in Figure 1. The first option would be putting a blend of blue, green, and red broadband-emitting phosphors on near-UV LED. There are many blue, green, and red broadband-emitting phosphors doped with Eu^2+^ or Ce^3+^ ions reported in the literature and probably the most well-known are blue-emitting BaMgAl_10_O_17_:Eu^2+^ (BAM) [4], green-emitting Lu_3_Al_5_O_12_:Ce^3+^ (LuAG:Ce) [5], and red-emitting CaAlSiN_3_:Eu^2+^ [6] and (Ca,Sr,Ba)_2_Si_5_N_8_:Eu^2+^ [7]. Due to presence of the broad emission band of the red-emitting nitride phosphor, part of it extends to wavelengths longer than 650 nm, i.e., to the spectral region where the human eye sensitivity is very low. This reduces the luminous efficacy of the whole light source, thus this part of the spectrum can be considered as a waste [8].

From the practical point of view, the white light source containing a red line emitting phosphor at ca. 610–615 nm is the best compromise between luminous efficacy and colour rendering [9]. Such concept for generating white light is shown in Figure 1b. For such a concept, Eu^3+^-doped phosphors seem to be the perfect candidates to replace the broadband red-emitting phosphors, since they exhibit strong luminescence in the range of 600–625 nm originating from intraconfigurational ^5^D_0_ → ^7^F_2_ transitions of Eu^3+^ ions. Moreover, Eu^3+^-doped phosphors also possess high photostability, high luminous efficacy and high quantum efficiency [9].

However, the main disadvantage of Eu^3+^-doped phosphors is weak absorption of Eu^3+^ ion in the blue or even in the near-UV. This is especially true for the excitation in the blue where only one ^7^F_0_ → ^5^D_2_ absorption transition is observed for Eu^3+^ ions. A bit more generous situation is for excitation in the near-UV. In this range the absorption transitions of Eu^3+^ ions are more abundant, for instance ^7^F_0_ → ^5^D_4_; ^5^G_J_; ^5^L_J_. Furthermore, in host matrices with low-lying charge transfer (CT) bands, these absorption transitions become relatively strong. Such matrices include molybdates [10], tungstates [11], vanadates [12], niobates [13], tantalates [14], etc.

Since europium and most of the other lanthanides are rather expensive, significant research is ongoing in finding lanthanide-free luminescent materials, especially emitting in the red spectral region. The main focus is placed on Mn^4+^ ions doped into inorganic matrices, which exhibit strong red luminescence. The concept of generating white light with elimination of Eu^3+^-doped phosphor, which is replaced by red-emitting K_2_SiF_6_:Mn^4+^ phosphor is shown in Figure 1c. Mn^4+^-doped inorganic materials show efficient and sharp line red luminescence in the 600–750 nm spectral range. However, only phosphors based on fluoride host matrices (for instance, K_2_SiF_6_:Mn^4+^, BaTiF_6_:Mn^4+^) possess suitable emission for white LEDs, i.e., below 650 nm [3,15,16].

The last concept to produce white light by employing near-UV-emitting LED chip is using a single-phase white-light-emitting phosphor (see Figure 1d). The advantage of such an approach is that the white light is produced within one phosphor; therefore, this eliminates several problems such as thorough blending of different colour-emitting phosphors particles, reabsorption, and so on. However, there are also some problems relating these phosphors, such as colour shift at different temperatures and so on. Most of the white-light-emitting phosphors are based on energy transfer between Ce^3+^ → Mn^2+^, Eu^2+^ → Mn^2+^, Ce^3+^ → Tb^3+^, Tb^3+^ → Eu^3+^ ion pairs or even more complicated systems [3,17].

In this contribution, the red-emitting Ba_2_Y_5_B_5_O_17_:Eu^3+^ phosphors were prepared by high temperature solid-state reaction. The optical properties of obtained luminescent materials were analysed with respect to excitation wavelength, Eu^3+^ concentration, and temperature. The ceramic samples from powders were also prepared in order to investigate possible application as a remote red-emitting phosphor.

## 2. Materials and Methods

Ba_2_Y_5_B_5_O_17_:Eu^3+^ phosphor powders were prepared by two-step solid state synthesis. Firstly, the stoichiometric amounts of raw materials (BaCO_3_ (99+%) and H_3_BO_3_ (99+%) from Acros Organics (Geel, Belgium), Y_2_O_3_ (99.99%) and Eu_2_O_3_ (99.99%) from Tailorlux (Münster, Germany)) were weighed and blended in an agate mortar with few millilitres of acetone to speed up the homogenization process. When all the acetone evaporated, the dry blend of starting materials was placed to the porcelain crucible and annealed at 450 °C for 4 h in air. Then the powder was reground and sintered at 1200 °C for 8 h in air. Finally, the sintered compounds were again ground to fine powder and used for further measurements. The Eu^3+^ concentration in given compounds was 0% (undoped sample), 1%, 5%, 10%, 25%, and 50%. Attempts to synthesize compounds with 75% and 100% Eu^3+^ were not successful.

Ba_2_Y_5_B_5_O_17_:50%Eu^3+^ phosphor powder was also pressed to ceramic disks with thicknesses of 0.73, 0.98, and 1.20 mm by applying 30 kN force for 5 min and, subsequently, annealing at 1200 °C for 5 h in air. The diameter of prepared ceramic disks was 8 mm.

X-ray diffraction (XRD) measurements were performed using Rigaku Ultima IV diffractometer (Rigaku, Tokyo, Japan) equipped with Cu anticathode, Soller slits to limit the divergence of X-ray beam and a nickel foil filter to attenuate the Cu K_β_ line. XRD patterns were recorded in the range of 15°–80° (scan rate was 0.05°/min) using the Bragg–Brentano configuration.

Field-emission Hitachi SU-70 Scanning Electron Microscope (SEM) machine (Hitachi, Tokyo, Japan) was used to investigate phosphor particle size and morphological features.

IR spectra were recorded in the range of 3000–400 cm^−1^ using Bruker Alpha Fourier-Transform Infrared (FTIR) spectrometer (Bruker, Ettlingen, Germany) with 4 cm^−1^ resolution.

Reflection spectra, room temperature and temperature dependent (77–500 K range) excitation and emission spectra, as well as Photoluminescence (PL) decay curves were recorded using Edinburgh Instruments FLS980 spectrometer (Edinburgh Instruments, Livingston, UK). The detailed description of the entire experimental setup is given in our previous publication [10].

The quantum efficiencies (*QE*) of the synthesized phosphors were determined by integrating sphere method. The equation summarizing the experiment can be written as [10]:

(1) $$QE=\frac{\int I_{em,sample}-\int I_{em,BaSO_{4}}}{\int I_{ref,BaSO_{4}}-\int I_{ref,sample}}\times 100\%=\frac{N_{em}}{N_{abs}}\times 100\%$$

here $\int I_{em,sample}$ and $\int I_{em,BaSO_{4}}$ denote the intensity of integrated emission of the sample and barium sulfate, respectively. $\int I_{ref,sample}$ and $\int I_{ref,BaSO_{4}}$ stand for the integrated reflectance of the sample and barium sulfate BaSO_4_, respectively. *N_em_* and *N_abs_* are the number of emitted and absorbed photons, respectively. Each measurement was repeated five times in order to get some statistical data.

## 3. Results and Discussion

The Ba_2_Y_5_B_5_O_17_ crystal structure was first reported by Hermus et al. in 2016 [18]. The compound crystallizes in primitive orthorhombic crystal structure and adopts *Pbcn* (#60) space group. There are four formula units per unit cell (Z = 4) and lattice parameters are: a = 17.38257 Å, b = 6.65299 Å, and c = 13.03055 Å.

**Figure 2:**
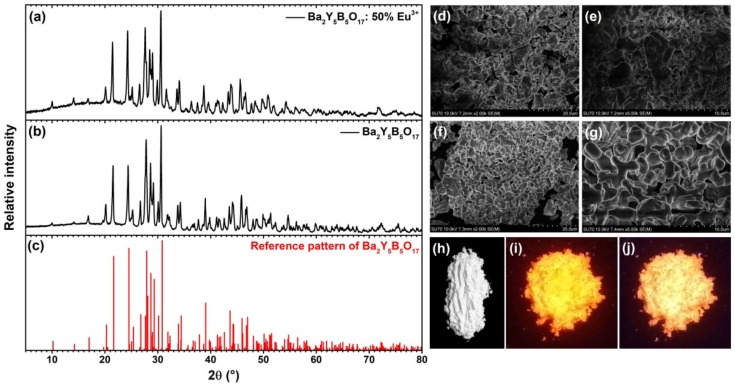
XRD patterns of Ba_2_Y_5_B_5_O_17_:50%Eu^3+^ (**a**), Ba_2_Y_5_B_5_O_17_ (**b**), and reference pattern of Ba_2_Y_5_B_5_O_17_ (**c**). SEM images of Ba_2_Y_5_B_5_O_17_:50%Eu^3+^ (**d**,**e**), and Ba_2_Y_5_B_5_O_17_ (**f**,**g**). Digital photographs of Ba_2_Y_5_B_5_O_17_:50%Eu^3+^ at daylight (**h**), and under 254 nm (**i**) and 365 nm (**j**) excitation.

Powder XRD patterns of synthesized Ba_2_Y_5_B_5_O_17_:50%Eu^3+^ and undoped Ba_2_Y_5_B_5_O_17_ are given in Figure 2a,b, respectively. The broad background is due to glass sample holder. The reference pattern is shown in Figure 2c. Single phase compounds isostructural with Ba_2_Y_5_B_5_O_17_ were synthesized when Eu^3+^ concentration has not exceeded 50%. The increase of Eu^3+^ concentration to 75% resulted in appearing of additional diffraction peaks indicating the presence of a mixture of phases. Furthermore, for 100% substituted compound the phase with *Pbcn* space group is lost in favour to a mixture of phases, which are difficult to identify. Nevertheless, some diffraction peaks could be attributable to an isostructural structure (with *Pnma* space group) to Ba_3_Pr_2_B_4_O_12_ compound [19].

SEM technique was employed to determine the particle size and form of the synthesized materials (see Figure 2d–g). The is virtually no changes in particle size and morphology with increasing Eu^3+^ concentration. The obtained particles are of irregular shape and formed from agglomerated crystallites.

The body colour of all synthesized Ba_2_Y_5_B_5_O_17_:Eu^3+^ was white regardless the Eu^3+^ concentration. The digital photograph of Ba_2_Y_5_B_5_O_17_:50%Eu^3+^ taken under daylight is depicted in Figure 2h. Nevertheless, this sample excited at both 254 and 365 nm radiation showed bright red luminescence as shown in Figure 2i,j, respectively.

**Figure 3:**
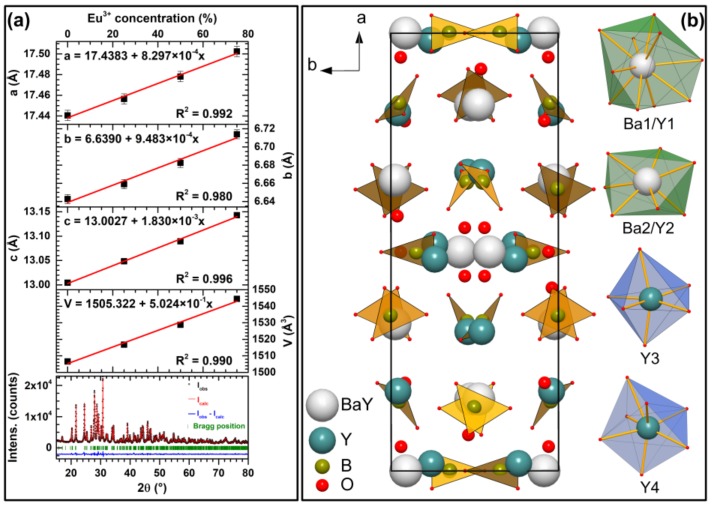
Unit cell parameters of Ba_2_Y_5_B_5_O_17_:Eu^3+^ as a function of Eu^3+^ concentration derived from the Le Bail fit and graphical representation of Ba_2_Y_5_B_5_O_17_ Le Bail fit (**a**). Unit cell of Ba_2_Y_5_B_5_O_17_ along the c-axis and coordination polyhedrons that Eu^3+^ ions could possibly occupy (**b**).

In Figure S1, which shows a zoom on the 18°–32° angular range, we can observe a displacement of the diffraction peaks towards the smaller angles indicating an increase of the lattice parameters with the insertion of Eu^3+^ ions. This evolution is not surprising because the ionic radius of Eu^3+^ (r(Eu^3+^)^VII^ = 1.01 Å) is larger than of Y^3+^ (r(Y^3+^)^VII^ = 0.96 Å) [20]. The VII coordination is used for the occupied site because Hermus et al. showed that the crystallographic sites occupied by Y^3+^ mainly have this coordination and the site with X coordination is mainly occupied by Ba^2+^ [18]. This was confirmed by calculated lattice parameters from XRD data employing the Le Bail fit, which showed that lattice parameters increase linearly with increasing Eu^3+^ concentration, which is also consistent with Vegard’s law [21] and confirms successful Eu^3+^ incorporation in this compound. The obtained linear evolution of lattice parameters together with graphical representation of Le Bail fit for Ba_2_Y_5_B_5_O_17_ compound is given in Figure 3a.

Figure 3b shows the unit cell of Ba_2_Y_5_B_5_O_17_ along c-axis together with coordination polyhedrons of sites that Eu^3+^/Y^3+^ can occupy. There are two sites that Y^3+^ shares with Ba^2+^ ions. The first is 10-coordinated and is mostly occupied by Ba^2+^ ions (Ba1/Y1). The second is mostly occupied by Y^3+^ ions and is seven-coordinated, forming a distorted pentagonal bipyramid (Ba2/Y2). There are also two independent sites that are occupied solely by Y^3+^ ions. One of them is six-coordinated forming distorted octahedron (Y3), whereas the other one is seven-coordinated and forms distorted capped trigonal prism (Y4). Boron atoms are three-coordinated and form trigonal planar units that are slightly distorted [18].

The IR spectra of Ba_2_Y_5_B_5_O_17_:Eu^3+^ compounds as a function of Eu^3+^ concentration are given in Figure S2. The spectra up to 75% Eu^3+^ look very similar. There are several strong absorption band in the range of 1400–400 cm^−1^. The most intensive absorption bands lie at 1195, 738, 620, 520, and 435 cm^−1^. Nevertheless, the IR spectra are relatively simple, since there are only BO_3_ units in the structure. The lines at 1400–1100 cm^−1^ are assigned to asymmetric stretching of BO_3_ groups whereas those in the range of 750–400 cm^−1^ to the ring bending [22,23].

**Figure 4:**
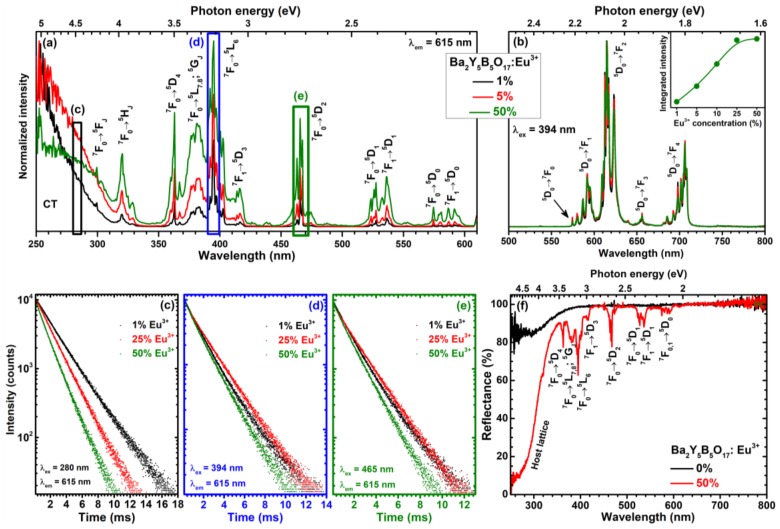
Eu^3+^ concentration dependent excitation (λ_em_ = 615 nm) (**a**) and emission (λ_ex_ = 394 nm) (**b**) spectra of Ba_2_Y_5_B_5_O_17_:Eu^3+^ phosphors. Inset in (**b**) shows integrated emission intensity as a function of Eu^3+^ concentration. PL decay curves (λ_em_ = 615 nm) of samples doped with 1%, 25%, and 50% Eu^3+^ recorded under different excitation wavelengths: 280 nm (**c**), 394 nm (**d**), and 465 nm (**e**). Reflection spectra of undoped and 50% Eu^3+^-doped Ba_2_Y_5_B_5_O_17_ specimens (**f**).

The excitation spectra of Ba_2_Y_5_B_5_O_17_:Eu^3+^ compounds doped with 1%, 5%, and 50% Eu^3+^ ions are given in Figure 4a. There is a broad excitation band ranging from 250 to 320 nm, which can be attributed to charge transfer transition (CT). At longer wavelengths, the typical Eu^3+^ excitation lines are visible. The excitation originates from the ^7^F_0_ and/or ^7^F_1_ ground state levels and ends up at ^5^F_J_ (ca. 320 nm), ^5^H_J_ (ca. 330 nm), ^5^D_4_ (ca. 363 nm), ^5^L_7,8_; ^5^G_J_ (ca. 370–390 nm), ^5^L_6_ (ca. 390–405 nm), ^5^D_3_ (ca. 410–420 nm), ^5^D_2_ (ca. 455–480 nm), ^5^D_1_ (ca. 515–545 nm), and ^5^D_0_ (ca. 570–600 nm) terminal levels [24,25]. The interesting feature of the given excitation spectra are the abundance of excitation lines, especially for the ^7^F_0_ → ^5^D_2_ transition. This originates from the fact that Eu^3+^ can occupy four lattice sites with each giving lines at slightly different wavelengths due to the different crystal field generated. This is the opposite to phosphors where only one lattice site is available for Eu^3+^ ions and there are very few excitation lines which, in turn, are also very narrow [13,26,27,28,29]. The absorption strength of the synthesized phosphors increased with increasing Eu^3+^ concentration. Since there are more excitation lines in excitation spectra, each of them could be used to excite the Ba_2_Y_5_B_5_O_17_:Eu^3+^ phosphor what is beneficial for practical application.

Figure 4b shows the emission spectra of Ba_2_Y_5_B_5_O_17_:Eu^3+^ samples doped with 1%, 5%, and 50% of Eu^3+^ ions for 394 nm excitation. There are five sets of lines visible in the given emission spectra. These emission lines originate from transitions starting form ^5^D_0_ excited state to ^7^F_0_ (ca. 577–581nm), ^7^F_1_ (ca. 582–600 nm), ^7^F_2_ (ca. 600–635 nm), ^7^F_3_ (ca. 655), and ^7^F_4_ (ca. 680–715 nm) terminal levels. It also evident the profile of emission spectra for 394 nm excitation is the same regardless the Eu^3+^ concentration. The strongest emission intensity was observed for Ba_2_Y_5_B_5_O_17_:50%Eu^3+^ sample as shown in the inset of Figure 4b.

Figure 4c–d shows PL decay curves (^5^D_0_ → ^7^F_2_ transition, λ_em_ = 615 nm) of Ba_2_Y_5_B_5_O_17_:Eu^3+^ samples doped with 1%, 25%, and 50% Eu^3+^ under excitation with 280, 394, and, 465 nm radiation, respectively. The PL of all samples decays bi-exponentially (except for 280 nm excitation where mono-exponential PL decay was observed until 25% Eu^3+^ concentration). The PL lifetime values for all samples were calculated according the following equation:

(2) $$I(t)=A+B_{1}e^{-\frac{t}{\tau_{1}}}+B_{2}e^{-\frac{t}{\tau_{2}}}$$

here *I(t)*, *A*, *B*_1_ and *B*_2_, *τ*_1_ and *τ*_2_ stand for PL intensity at a given time *t*, background, constants, and PL lifetime values, respectively. The calculated PL lifetime values together with standard deviations, relative percentage and calculated average PL lifetime values for 280, 394, and 465 nm excitation are tabulated in Tables S1–S3, respectively. Under 280 nm excitation, the average PL lifetime values gradually decrease from 2400 to 1440 μs when changing Eu^3+^ concentration from 1% to 50%. Slightly different PL lifetime values were obtained under 394 and 465 nm excitation. Here average PL lifetime values increased from ca. 1600 μs (1% Eu^3+^-doped sample) to ca. 1760 μs (25% Eu^3+^-doped sample) and then again decreased to ca. 1390 μs when Eu^3+^ concentration reached 50%.

The reflection spectra of undoped Ba_2_Y_5_B_5_O_17_ and Ba_2_Y_5_B_5_O_17_:50%Eu^3+^ are depicted in Figure 4f. The body colour of Ba_2_Y_5_B_5_O_17_ powder was white suggesting that this material does not absorb visible light. This goes hand in hand with the respective reflection spectrum where slight absorption starts at wavelengths shorter than 380 nm. The reflection spectrum of Ba_2_Y_5_B_5_O_17_:50%Eu^3+^ specimen, in turn, contains several sets of absorption lines in the visible light range and broad absorption band starting at ca. 340 nm and strongly increasing going to shorter wavelengths. This spectrum is very similar to excitation spectra of the given compounds; therefore, the assigned transitions are also the same for both.

**Figure 5:**
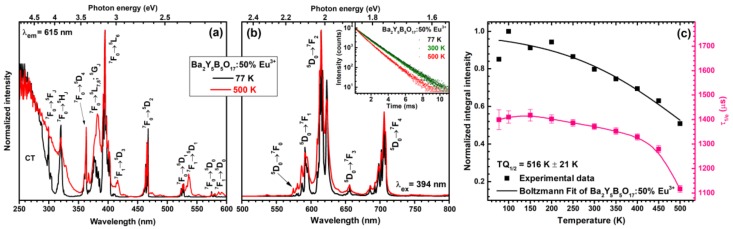
Excitation (λ_em_ = 615 nm) (**a**) and emission (λ_ex_ = 394 nm) (**b**) spectra of Ba_2_Y_5_B_5_O_17_:50%Eu^3+^ at 77 and 500 K temperatures. Inset in (**b**) shows temperature dependent PL decay curves (λ_ex_ = 394 nm, λ_em_ = 615 nm) of Ba_2_Y_5_B_5_O_17_:50%Eu^3+^. Temperature dependent emission integrals with Boltzmann fit and PL lifetime values (**c**).

The excitation spectra (for 615 nm emission) of Ba_2_Y_5_B_5_O_17_:50%Eu^3+^ sample were also measured at 77 and 500 K temperatures. These spectra are depicted in Figure 5a. Despite the similar profile of both spectra there are also some significant changes going on when temperature increases from 77 to 500 K. First of all, at low temperatures the thermal population of ^7^F_1_ is very suppressed; therefore, mostly the lines originating from the ^7^F_0_ ground state transitions to various excited states are visible. This is best observed for ^7^F_1_ → ^5^D_3_ (ca. 415 nm), ^7^F_1_ → ^5^D_1_ (ca. 535 nm) and ^7^F_1_ → ^5^D_0_ (ca. 590 nm) transitions, which do not occur at 77 K but are relatively strong at 500 K temperature. Moreover, the increase of the width of charge transfer state is also evident at elevated temperatures. This phenomenon was also observed by other researchers for different types of compounds doped with Eu^3+^ ions [30,31]. The broadening of the CT band can be explained as follows; at low temperatures transition to the CT state starts from one vibrational level of the ground state (ν_1_); however, at elevated temperatures this transition starts at the higher vibrational levels of the ground state. Therefore, the absorption transition energy decreases leading to the red shift of CT band [31].

Figure 5b shows normalized emission spectra (λ_ex_ = 394 nm) of Ba_2_Y_5_B_5_O_17_:50%Eu^3+^ sample recorded at 77 and 500 K temperatures. Both spectra are nearly identical and the only observable difference is broader emission lines at elevated temperature. The lattice vibrations increase with increasing temperature; thus, the local surrounding of Eu^3+^ ions changes. This yields slight changes in crystal field strength and emission of Eu^3+^ ions at somewhat different wavelengths what eventually leads to emission line broadening. Inset in Figure 5b demonstrates temperature dependent PL decay curves (λ_ex_ = 394 nm, λ_em_ = 615 nm) of Ba_2_Y_5_B_5_O_17_:50%Eu^3+^ specimen. The exact calculated PL lifetime values are summarized in Table S4. The PL decay curves become steeper at temperatures higher than 250 K showing decreasing PL lifetime values. This can be better appreciated from Figure 5c where PL lifetime values are plotted as a function of temperature. Severe decrease in PL lifetime values is observed when temperature reaches 450 K, indicating the increase of rates of nonradiative transitions.

Temperature dependent emission integrals can be used to evaluate phosphor performance at elevated temperatures. This is very important parameter since modern high power LEDs during operation can heat up to temperatures as high as 400 K and even above. The temperature dependent normalized emission integral values as a function of temperature are given in Figure 5c. These values were also used to determine the *TQ*_1/2_ value (temperature, when phosphor emission decreases twice) employing Boltzmann fit [32]. The extracted *TQ*_1/2_ value for Ba_2_Y_5_B_5_O_17_:50%Eu^3+^ phosphor sample was 516 ± 21 K. Moreover, at 450 K ca. 65% of initial emission still remains indicating relatively high emission stability with increasing temperature.

**Figure 6:**
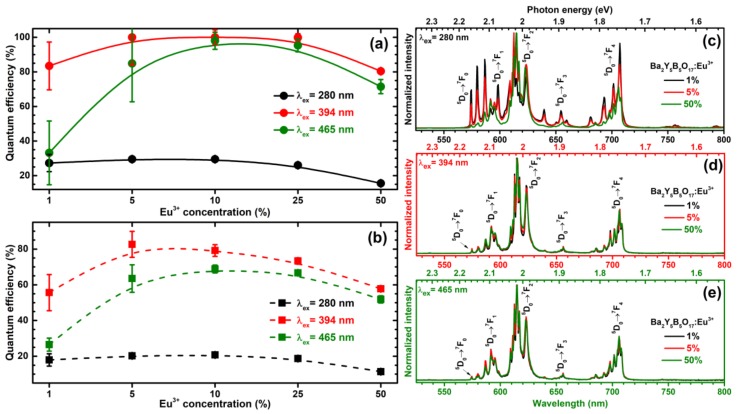
Quantum efficiencies of Ba_2_Y_5_B_5_O_17_:Eu^3+^ samples as a function of Eu^3+^ concentration and excitation wavelength for 500–800 nm range (**a**) and 500–650 nm range (**b**). Emission spectra of 1%, 5% and 50% Eu^3+^-doped specimens under 280 nm (**c**), 394 nm (**d**), and 465 nm (**e**) excitation wavelength.

The quantum efficiency (*QE*) is also very important parameter of the phosphor if it is considered for practical application. The quantum efficiency values of Ba_2_Y_5_B_5_O_17_:Eu^3+^ phosphors as a function of Eu^3+^ concentration and excitation wavelength are shown in Figure 6a (taking the whole emission integral from 500 to 800 nm). Excitation at 280 nm yielded low quantum efficiencies ca. 30% for samples doped with 5% and 10% Eu^3+^. Further increase of Eu^3+^ concentration resulted in gradual decrease of *QE* to ca. 15% for 50% Eu^3+^-doped specimen. The low quantum efficiency for 280 nm (CT band) can be explained by the nonradiative relaxation of the excited electron through the charge transfer state [24,33]. Much higher *QE* values were obtained if 394 and 465 nm radiation was used for sample excitation. In this case *QE* vales as high as ca. 100% were obtained for samples doped with 10% and 25% Eu^3+^. The increase of Eu^3+^ concentration to 50% led to decrease of *QE* to ca. 80% for 394 nm excitation and ca. 72% for 465 nm excitation. Moreover, since the human eye is very insensitive to wavelengths above 650 nm [8], we have also calculated the “effective” quantum efficiency where we discarded the emission above 650 nm. The obtained values are given in Figure 6b. *QE* values calculated in this way are, of course, lower than those for which the whole emission was taken into account. However, the overall trend remains the same. The highest *QE* values are obtained for samples doped with 5–25% Eu^3+^. For 280 nm excitation the highest “effective” *QE* was ca. 20% (5% and 10% Eu^3+^-doped samples); for 395 nm excitation ca. 80% (5% and 10% Eu^3+^-doped samples); for 465 nm excitation ca. 65% (5–25% Eu^3+^-doped samples). These results show that the synthesized phosphors have potential to be used in both near-UV and blue InAlGaN chip-driven white LEDs.

We have also recorded the emission spectra (see Figure 6b–d) of Ba_2_Y_5_B_5_O_17_:Eu^3+^ samples under 280 nm (CT transition) and 465 nm (^7^F_0_ → ^5^D_2_ transition) excitation and observed some changes in emission spectra profile. Excitation with both 394 nm (Figure 6d) and 465 nm (Figure 6e) wavelength radiation yields virtually identical emission spectra. However, this is not the case for 280 nm excitation as shown in Figure 6c. Here, only the sample with 50% of Eu^3+^ gives emission spectra similar to those recorded under 394 and 465 nm excitation. Samples doped with low Eu^3+^ concentrations (1% and 5% in the given case), in turn, yield much different emission spectra, whereas lines originating from ^5^D_0_ → ^7^F_0_, ^5^D_0_ → ^7^F_1_, ^5^D_0_ → ^7^F_3_, and ^5^D_0_ → ^7^F_4_ transitions are much more pronounced. The explanation of such different emission spectra profile could be that, regardless the obtained single phase materials from the XRD data, there is still some small amount of impurity phases not detectable with XRD. These impurity phases would give their own emission spectra, which, of course, are different than for Ba_2_Y_5_B_5_O_17_:Eu^3+^. Thus, at low Eu^3+^ concentrations the emission intensity from these impurity phases would be rather comparable to Ba_2_Y_5_B_5_O_17_:Eu^3+^ emission. With increasing Eu^3+^ concentration, the concentration of impurities would remain the same; therefore, the emission from Ba_2_Y_5_B_5_O_17_:Eu^3+^ compounds would start to dominate the emission spectra. Nevertheless, the given two explanations are just assumptions and further spectroscopic investigation is needed in order to clarify this phenomenon.

In inorganic matrices the absorption of rare earth ions is relatively weak because the transitions within 4f orbitals are forbidden [34]. As was already mentioned, there are several classes of inorganic compounds, for instance, molybdates, tungstates, vanadates, etc., with low-lying charge transfer states, which spectrally overlap with energy levels of rare earth ions resulting in stronger emission due to energy transfer [35]. Preparation of ceramic discs from phosphor powder can also be one of the ways to increase the absorption efficiency due to increased penetration depth of the incident light. This approach also reduces the temperature of the phosphor since the ceramic layer is deposited further from hot InAlGaN chip [36]. To test this approach, we have prepared three ceramic disks (the thickness of disks was 0.73, 0.98, and 1.20 mm) from Ba_2_Y_5_B_5_O_17_:50%Eu^3+^ sample, placed them on 375, 400, and 455 nm emitting LEDs and measured emission spectra of the obtained light sources.

The digital image of 0.73-mm-thick ceramic disk is given in Figure S3a. Here we need to note, that the prepared ceramics are not transparent, but translucent instead. The image of ceramic disks with all three thicknesses under 365 nm excitation is shown in Figure S3b. Finally, the image of the 1.20-mm-thick Ba_2_Y_5_B_5_O_17_:50%Eu^3+^ ceramic disk illuminated with the 400 nm emitting LED from below is given in Figure S3c. The pinkish red colour was obtained due to mixing of the red light emitted by the ceramic disk and passed through unabsorbed light form 400 nm emitting LED.

**Figure 7:**
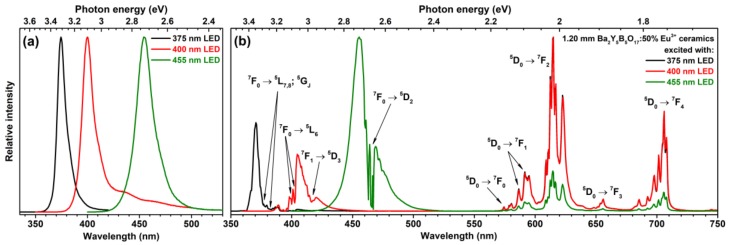
Emission spectra of 375, 400, and 455 nm emitting LEDs (**a**). Emission spectra of 1.20-mm-thick Ba_2_Y_5_B_5_O_17_:50%Eu^3+^ ceramic disk excited with 375, 400, and 455 nm emitting LEDs (**b**).

The emission spectra of sole LEDs are given in Figure 7a, whereas Figure 7b shows the emission spectra of these LEDs with the thickest Ba_2_Y_5_B_5_O_17_:50%Eu^3+^ ceramic disk on top. The absorption of the LED emitted light increased with increasing ceramic thickness; therefore, only 1.2 mm ceramic disk properties will be analyzed further. It is evident that 1.2-mm-thick ceramic disk is still not capable to absorb all the incident light of either investigated LED. However, this is not necessarily a drawback, since the unabsorbed incident light can be used for excitation of phosphors emitting other colours. It is also clear that the ceramic disks absorb light emitted by 400 nm most efficiently. This, however, is not surprising, since there are a lot of absorption lines of Eu^3+^ ions in this spectral region. The light emitted by 375 nm LED is also efficiently absorbed. In both cases the emission spectrum of the ceramic disk is identical. The worst absorption was observed of 455 nm LED emitted light. In this area there is only one absorption line originating from the ^7^F_0_ → ^5^D_2_ transition; therefore, most of light emitted by LED passes through the ceramic disk unabsorbed. However, the absorption of 455 nm emitting LED radiation is still stronger if compared to ceramic disks prepared from other materials [10,32,37]. This arises from the fact that, as was already discussed above, there are four different lattice in Ba_2_Y_5_B_5_O_17_ compound that Eu^3+^ can occupy; thus, simply, there are more lines originating from the same transition.

**Figure 8:**
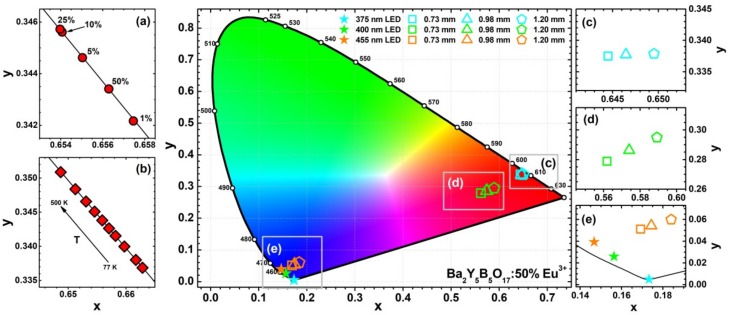
Magnified sections of CIE 1931 colour space diagram showing colour coordinates of Ba_2_Y_5_B_5_O_17_:Eu^3+^ samples as a function of Eu^3+^ concentration (λ_ex_ = 394 nm) (**a**) and temperature (λ_ex_ = 394 nm) (**b**) for Ba_2_Y_5_B_5_O_17_:50%Eu^3+^ specimen. Enlarged areas (**c**), (**d**), and (**f**) show colour coordinates of Ba_2_Y_5_B_5_O_17_:50%Eu^3+^ ceramic disks (0.73, 0.98, and 1.20 mm in thickness) excited with 375, 400, and 455 nm emitting LEDs, respectively.

The CIE 1931 colour coordinates and luminous efficacy (LE) values were calculated from the respective emission spectra. The fragments of CIE 1931 colour space with colour coordinates of Ba_2_Y_5_B_5_O_17_:Eu^3+^ as a function of Eu^3+^ concentration and temperature dependent colour coordinates of Ba_2_Y_5_B_5_O_17_:50%Eu^3+^ sample are given in Figure 8a,b, respectively. These colour coordinates are located directly on the edge of the CIE 1931 colour space diagram, indicating that the emission spectra are perceived as a monochromatic light by human eye. Colour coordinates also tend to move to more orange spectral region when Eu^3+^ concentration or phosphor temperature is increased. On the other hand, the shift is very small; thus colour coordinates can be considered as stable. The exact calculated values together with LE values as a function of Eu^3+^ concentration and excitation wavelength are summarized in Table S5. The LE values of all the samples are relatively the same and fluctuate around 240 lm/W_opt_. This could be expected, since emission spectra do not change much with increasing Eu^3+^ concentration and excitation wavelength. The largest change in LE values was observed if samples were excited with 280 nm. In this case, LE decreased from 250 lm/W_opt_ (for 1% Eu^3+^-doped sample) to 243 lm/W_opt_ (for 50% Eu^3+^-doped sample). This is in good agreement with emission spectra shown in Figure 6b. With increasing Eu^3+^ concentration, the intensity of lines (^5^D_0_ → ^7^F_1_ transition) in the orange spectral region decreases; thus the LE values decrease, because human eye is more sensitive to the orange light [38]. Moreover, the LE values of the synthesized Ba_2_Y_5_B_5_O_17_:50%Eu^3+^ phosphors are higher or very similar to the ones reported for some well-established red-emitting Eu^2+^-doped phosphors, namely, Sr_2_Si_5_N_8_:Eu^2+^ (λ_em_ = 620 nm; LE = 240 lm/W_opt_), CaAlSiN_3_:Eu^2+^ (λ_em_ = 650 nm; LE = 150 lm/W_opt_), and CaS:Eu^2+^ (λ_em_ = 650 nm; LE = 85 lm/W_opt_) [9]. However, they are slightly lower if compared to other Eu^3+^ phosphors possessing lower intensity of ^5^D_0_ → ^7^F_4_ transition ca. 700 nm, for instance, Li_3_Ba_2_Eu_3_(MoO_4_)_8_ (λ_em_ = 615 nm; LE = 312 lm/W_opt_) [39] or LiLa(MoO_4_)_2_:Eu^3+^ (λ_em_ = 616 nm; LE = 280 lm/W_opt_) [40].

The CIE 1931 chromaticity coordinates for light sources obtained by combining 375, 400, and 455 nm emitting LEDs with various thicknesses of Ba_2_Y_5_B_5_O_17_:50%Eu^3+^ ceramic disks were also calculated and presented in Figure 8c–e, respectively. When 375 nm LED is used for excitation, the red light source is obtained with colour coordinates located close to the edge of the chromaticity diagram. However, this is not the case for other two LEDs used for excitation. Combination of 400 nm emitting LED with Ba_2_Y_5_B_5_O_17_:50%Eu^3+^ ceramic disks yielded light sources emitting in the purple region due to presence of red light emitted by ceramics and unabsorbed violet light emitted by LED. The colour coordinates shifted towards red region if the thickness of ceramics was increased. Similarly, only the tints of blue light were obtained if 455 nm LED was used to excite the ceramic disks, because only very small fraction of 455 nm LED emission was absorbed by ceramic disks regardless their thickness. This clearly shows, that 455 nm emitting LED is not suitable for excitation of these materials. The exact calculated CIE 1931 colour coordinates together with LE values are given in Table S7. The calculated LE values are relatively the same for the light sources obtained by combining Ba_2_Y_5_B_5_O_17_:50%Eu^3+^ ceramic disks with 375 and 400 nm emitting LEDs. The LE values increase from ca. 150 to ca. 185 lm/W_opt_ for the thinnest and the thickest ceramics, respectively. This is related to decreasing emission fraction of LED to which human eye is extremely insensitive. Furthermore, even lower LE values (ca. 70 lm/W_opt_) were obtained if 455 nm emitting LED was used to excite ceramic disks. As mentioned above, only a small fraction of this LED is absorbed by the ceramic disk. This results in a situation when emission spectra of the light source contain the strongest emission in the blue and red spectral regions. Human eye is not very sensitive in either of these regions and thus the LE values of such light source are extremely low. Similar results were also reported earlier for ceramic disks prepared from other compounds [10,32,37].

## 4. Conclusions

Phase pure Ba_2_Y_5_B_5_O_17_:Eu^3+^ red-emitting phosphors were prepared by high temperature solid state reaction method. The solubility of Eu^3+^ in the given host matrix was found to be 50% with respect to Y^3+^ ions. All samples exhibited bright red luminescence if excited with UV, near-UV and blue radiation. The emission spectra were dominated by ^5^D_0_ → ^7^F_2_ and ^5^D_0_ → ^7^F_4_ transitions of Eu^3+^ ions at ca. 615 and 705 nm, respectively. The synthesized phosphors possess high colour purity and luminous efficacy values (ca. 240 lm/W_opt_). Moreover, the 50% Eu^3+^-doped sample lost half of the efficiency only at ca. 500 K, showing high thermal stability. The prepared Ba_2_Y_5_B_5_O_17_:50%Eu^3+^ ceramic disks showed increasing absorption of near-UV and blue radiation with increasing thickness of the ceramic disk. It was also observed that near-UV radiation is more efficiently absorbed by ceramic disks if compared to the blue radiation. This is due to more abundant absorption transitions of Eu^3+^ ions in this spectral area. Finally, samples doped with 5%, 10%, and 25% Eu^3+^ showed quantum efficiency close to 100% if excited with 394 nm radiation what is a huge benefit for practical application of the synthesized phosphors. The “effective” *QE* values, obtained by discarding emission above 650 nm, were as high as 80% for 394 nm excitation.

## Supplementary Materials

The following are available online at https://www.mdpi.com/1996-1944/13/3/763/s1, Figure S1: XRD patterns of Ba_2_Y_5_B_5_O_17_: Eu^3+^ phosphors, Figure S2: FTIR spectra of Ba_2_Y_5_B_5_O_17_: Eu^3+^ phosphors, Figure S3. Digital images of: 0.73-mm-thick Ba_2_Y_5_B_5_O_17_:50%Eu^3+^ ceramic disk under daylight (a); 0.73, 0.98, and 1.20-mm-thick (from left to right) Ba_2_Y_5_B_5_O_17_:50%Eu^3+^ ceramic disks under 365 nm excitation (b); 1.20-mm-thick on top of 400 nm emitting LED (c), Table S1: PL lifetime values of Ba_2_Y_5_B_5_O_17_:Eu^3+^ phosphors as a function of Eu^3+^ concentration (λ_ex_ = 280 nm, λ_em_ = 615 nm), Table S2: PL lifetime values of Ba_2_Y_5_B_5_O_17_:Eu^3+^ phosphors as a function of Eu^3+^ concentration (λ_ex_ = 394 nm, λ_em_ = 615 nm), Table S3: PL lifetime values of Ba_2_Y_5_B_5_O_17_:Eu^3+^ phosphors as a function of Eu^3+^ concentration (λ_ex_ = 465 nm, λ_em_ = 615 nm), Table S4: PL lifetime values of Ba_2_Y_5_B_5_O_17_:50%Eu^3+^ as a function of temperature (λ_ex_ = 394 nm, λ_em_ = 615 nm), Table S5: CIE 1931 colour coordinates and luminous efficacies (LE) of synthesized phosphors as a function of Eu^3+^ concentration and excitation wavelength, Table S6: CIE 1931 colour coordinates and luminous efficacies (LE) of Ba_2_Y_5_B_5_O_17_:50%Eu^3+^ as a function of temperature (λ_ex_ = 394 nm), Table S7: CIE 1931 colour coordinates and luminous efficacies (LE) of different thicknesses Ba_2_Y_5_B_5_O_17_:50%Eu^3+^ ceramics mounted on 375, 400, and 455 nm LEDs.
