# Supplementary material for: Temperature-Dependent Luminescence of Red-Emitting Ba2Y5B5O17: Eu3+ Phosphors with Efficiencies Close to Unity for Near-UV LEDs
Source: Materials (Basel). 2020 Feb 7;13(3):763. doi: 10.3390/ma13030763 (PMC7040723; doi:10.3390/ma13030763)
Supplement: Supplementary file 1 [file materials-13-00763-s001.pdf]

materials

MDPI

Supplementary Materials: Temperature-Dependent Luminescence of Red-Emitting $Ba_2Y_5B_5O_{17}$: Eu^{3+} Phosphors with Efficiencies Close to Unity for Near-UV LEDs

Egle Ezerskyte, Julija Grigorjevaite, Agne Minderyte, Sebastien Saitzek and Arturas Katelnikovas

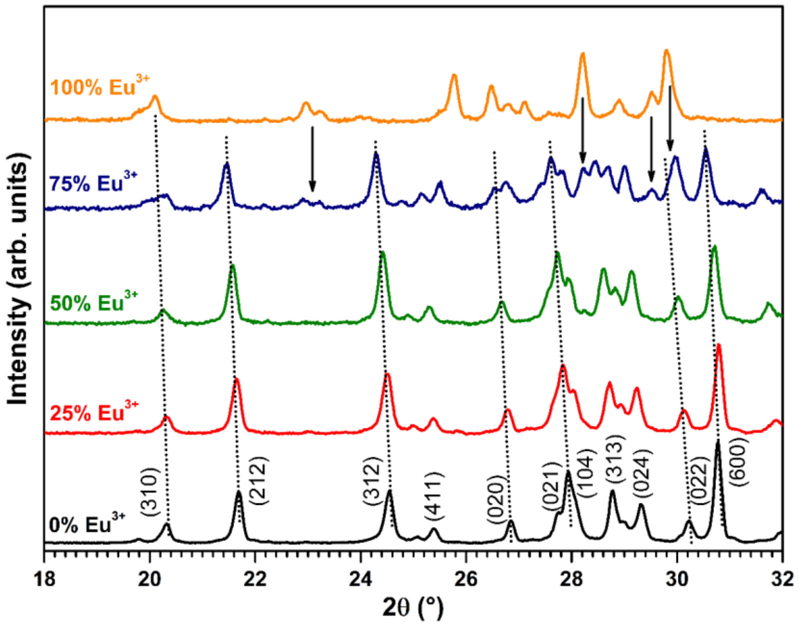

Figure S1. XRD patterns of $Ba_2Y_5B_5O_{17}$: Eu^{3+} phosphors.

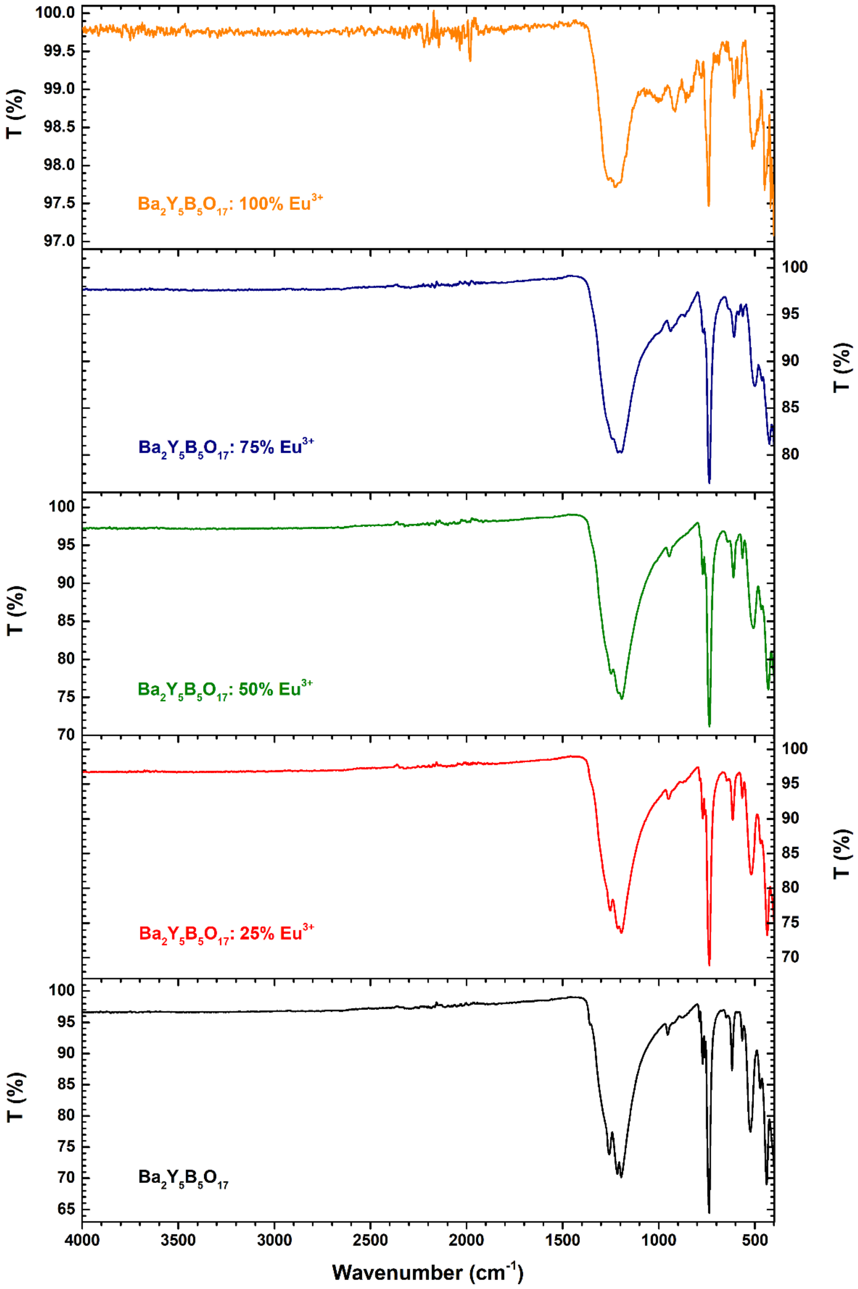

Figure S2. FTIR spectra of $Ba_2Y_5B_5O_{17}$: Eu^{3+} phosphors.

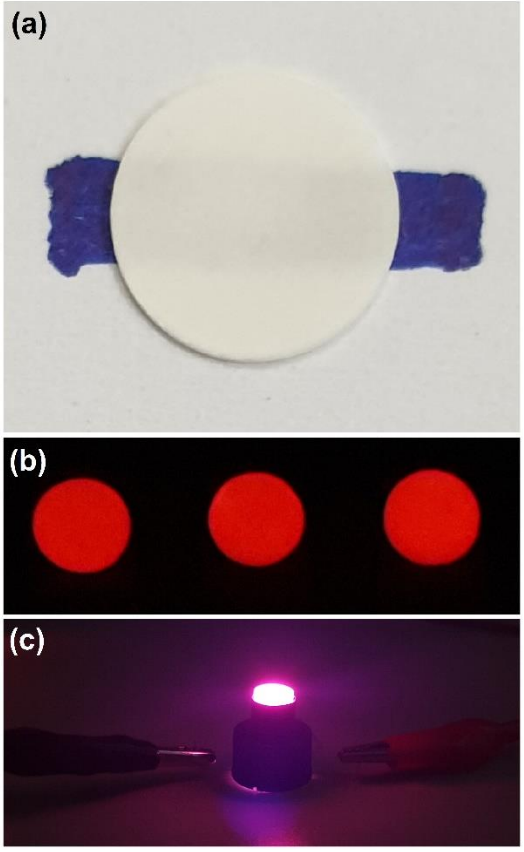

Figure S3. Digital images of: 0.73-mm-thick $Ba_2Y_5B_5O_{17}$:50%Eu^{3+} ceramic disk under daylight (a); 0.73, 0.98, and 1.20-mm-thick (from left to right) $Ba_2Y_5B_5O_{17}$:50%Eu^{3+} ceramic disks under 365 nm excitation (b); 1.20-mm-thick on top of 400 nm emitting LED (c).

Table S1. PL lifetime values of $Ba_2Y_5B_5O_{17}$:Eu^{3+} phosphors as a function of Eu^{3+} concentration (λ_{ex} = 280 nm, λ_{em} = 615 nm).

Eu^{3+} (%)	τ_1 (μs)	Rel. %	τ_2 (μs)	Rel. %	$\overline{\tau}_{1/e}$ (μs)
**1**	2400 ± 2	100	–	–	–
**5**	2290 ± 2	100	–	–	–
**10**	2140 ± 2	100	–	–	–
**25**	1850 ± 2	100	–	–	–
**50**	920 ± 34	22	1600 ± 16	78	1450 ± 20

Table S2. PL lifetime values of $Ba_2Y_5B_5O_{17}$:Eu^{3+} phosphors as a function of Eu^{3+} concentration (λ_{ex} = 394 nm, λ_{em} = 615 nm).

Eu^{3+} (%)	τ_1 (μs)	Rel. %	τ_2 (μs)	Rel. %	$\overline{\tau}_{1/e}$ (μs)
**1**	1240 ± 11	69	2320 ± 39	31	1570 ± 20
**5**	1170 ± 18	44	2130 ± 22	56	1710 ± 20
**10**	1010 ± 22	25	2000 ± 12	75	1750 ± 15
**25**	880 ± 50	8	1840 ± 8	92	1760 ± 11
**50**	840 ± 30	20	1540 ± 11	80	1400 ± 15

Table S3. PL lifetime values of $Ba_2Y_5B_5O_{17}$:Eu^{3+} phosphors as a function of Eu^{3+} concentration (λ_{ex} = 465 nm, λ_{em} = 615 nm).

Eu^{3+} (%)	τ_1 (μs)	Rel. %	τ_2 (μs)	Rel. %	$\overline{\tau}_{1/e}$ (μs)
**1**	1230 ± 11	64	2320 ± 30	36	1620 ± 18
**5**	1160 ± 18	41	2130 ± 20	59	1730 ± 19

**10**	940 ± 20	22	1970 ± 10	78	1740 ± 12
**25**	690 ± 35	6	1830 ± 5	94	1760 ± 7
**50**	740 ± 27	15	1500 ± 8	85	1390 ± 11

Table S4. PL lifetime values of $Ba_2Y_5B_5O_{17}$:50%Eu^{3+} as a function of temperature (λ_{ex} = 394 nm, λ_{em} = 615 nm).

T (K)	τ_1 (μs)	Rel. %	τ_2 (μs)	Rel. %	$\overline{\tau}_{1/e}$ (μs)
**77**	1020 ± 83	21	1500 ± 29	79	1400 ± 41
**100**	940 ± 64	16	1500 ± 18	84	1410 ± 26
**150**	920 ± 52	18	1530 ± 17	82	1420 ± 23
**200**	830 ± 42	15	1500 ± 12	85	1400 ± 17
**250**	800 ± 32	17	1500 ± 10	83	1380 ± 14
**300**	740 ± 29	15	1480 ± 9	85	1370 ± 12
**350**	750 ± 28	17	1480 ± 10	83	1360 ± 13
**400**	750 ± 28	18	1450 ± 10	82	1320 ± 13
**450**	750 ± 27	21	1420 ± 12	79	1280 ± 15
**500**	670 ± 23	23	1250 ± 11	77	1120 ± 14

Table S5. CIE 1931 colour coordinates and luminous efficacies (LE) of synthesized phosphors as a function of Eu^{3+} concentration and excitation wavelength.

Eu^{3+} (%)	λ_{ex} = 280 nm			λ_{ex} = 394 nm			λ_{ex} = 465 nm		
	CIE 1931		LE (lm/W_{opt})	CIE 1931		LE (lm/W_{opt})	CIE 1931		LE (lm/W_{opt})
	x	y		x	y		x	y	
**1**	0.62257	0.37682	250	0.65746	0.34217	235	0.65064	0.34871	245
**5**	0.63546	0.36414	247	0.65504	0.34462	238	0.65214	0.34749	242
**10**	0.64174	0.35789	249	0.65407	0.34561	239	0.65196	0.34768	242
**25**	0.65166	0.34802	242	0.65397	0.34572	241	0.65366	0.34602	240
**50**	0.65458	0.34511	243	0.65629	0.34341	240	0.65642	0.34328	238

Table S6. CIE 1931 colour coordinates and luminous efficacies (LE) of $Ba_2Y_5B_5O_{17}$:50%Eu^{3+} as a function of temperature (λ_{ex} = 394 nm).

T (K)	CIE 1931		LE (lm/W_{opt})
	x	y	
**77**	0.66286	0.33686	227
**100**	0.66171	0.33800	228
**150**	0.65972	0.33998	231
**200**	0.65818	0.34152	234
**250**	0.65706	0.34264	235
**300**	0.65589	0.3438	235
**350**	0.65458	0.34509	236
**400**	0.65308	0.34657	237
**450**	0.65124	0.34838	238
**500**	0.64869	0.35086	239

Table S7. CIE 1931 colour coordinates and luminous efficacies (LE) of different thicknesses $Ba_2Y_5B_5O_{17}$:50%Eu^{3+} ceramics mounted on 375, 400, and 455 nm LEDs.

LED (nm)	Thickness (mm)	CIE 1931		LE (lm/W_{opt})
		x	y	
**375**	**0.73**	0.64451	0.33749	146
	0.98	0.64645	0.33774	166

	1.20	0.64953	0.33786	190
400	0.73	0.561s92	0.27882	155
	0.98	0.57402	0.28627	167
	1.20	0.58928	0.29480	180
455	0.73	0.16916	0.05116	69
	0.98	0.17442	0.05422	73
	1.20	0.18400	0.05984	80
